# Diversifying Emotional Dialogue Generation via Selective Adversarial Training

**DOI:** 10.3390/s23135904

**Published:** 2023-06-25

**Authors:** Bo Li, Huan Zhao, Zixing Zhang

**Affiliations:** College of Computer Science and Electronic Engineering, Hunan University, Changsha 410082, China; blee@hnu.edu.cn (B.L.); hzhao@hnu.edu.cn (H.Z.)

**Keywords:** dialog systems, emotional response generation, diversity enhancement, latent variables, conditional variational autoencoder

## Abstract

Emotional perception and expression are very important for building intelligent conversational systems that are human-like and attractive. Although deep neural approaches have made great progress in the field of conversation generation, there is still a lot of room for research on how to guide systems in generating responses with appropriate emotions. Meanwhile, the problem of systems’ tendency to generate high-frequency universal responses remains largely unsolved. To solve this problem, we propose a method to generate diverse emotional responses through selective perturbation. Our model includes a selective word perturbation module and a global emotion control module. The former is used to introduce disturbance factors into the generated responses and enhance their expression diversity. The latter maintains the coherence of the response by limiting the emotional distribution of the response and preventing excessive deviation of emotion and meaning. Experiments are designed on two datasets, and corresponding results show that our model outperforms existing baselines in terms of emotional expression and response diversity.

## 1. Introduction

Building dialogue systems with the ability to communicate naturally with people is a fundamental task of building intelligent agents. Emotional expression is a key characteristic of a human-like dialogue system. Enabling dialogue systems to understand and express emotions has multiple benefits [1,2]:

- More natural communication: Emotions are an important part of human communication. When dialogue systems can understand and express emotions, they can more accurately capture and respond to users’ emotional expressions, making conversations more natural and human.
- Emotion recognition: By understanding the user’s emotions, the dialogue system can better understand the user’s intentions and needs. Emotion recognition helps to parse user input more precisely and provide responses and support based on emotional information.
- Emotional support: The dialogue system can express emotions and provide users with emotional support and emotional management. When users need reassurance, encouragement, or understanding, the emotional expression of dialogue systems can provide a positive impact and emotional connection.
- Improvement of user experience: Emotion plays an important role in user experience. When the dialog system is able to recognize and respond to the user’s emotions, the user feels understood and cared for, which helps to build a better user experience and enhance user satisfaction with the dialog system.
- Emotion research and application: The ability of dialogue systems to understand and express emotions also contributes to the field of emotion research and application. For example, in research on affective computing, affective analysis, and affective intelligence, dialogue systems can provide an experimental platform and tool.

Early approaches relied on artificially designed rules to generate an emotional response from the system, but these methods had significant shortcomings in terms of cost and flexibility. Deep neural networks have greatly advanced due to their development and recent research has achieved promising results in this area [3,4,5,6]. On one hand, these advancements have benefited from the successful application of general models such as seq2seq, CVAE, and transformers in the task of generating dialogue responses. This has significantly enhanced the performance of the models. On the other hand, the increasing focus on affective computing within the academic community has led to the development of affective dialogue datasets such as Emotional Chatting Machines [3] and Empathetic Dialogues [7]. These datasets provide valuable data support for model training and learning.

Another important capability that a dialogue system should possess is the ability to generate diverse responses. However, one problem with neural training approaches is that the resulting models tend to generate high-frequency responses, often providing meaningless statements such as “I don’t know”. This problem arises because of the MLE training target, leading to an overconfident probability estimate for high-frequency tokens [8], which results in a decrease in diversity [9,10]. As dialogue is a one-to-many mapping, multiple responses are appropriate for the same input. Therefore, the ideal training target should be a soft target that assigns probability weights to multiple valid candidates [11]. However, studies have shown that the distribution of real text fluctuates significantly in the confusion degree of each target, and it is hard to achieve soft targets [12].

To tackle the problems mentioned above, we propose introducing perturbations to the decoding process of the system, which can reduce the generation of high-frequency words to some extent. In order to maintain emotional balance, we use the response’s emotion label to regulate the impact of perturbations on the system’s output. This ensures that the generated response maintains emotional consistency, preventing large deviations that could disrupt the overall emotional context.

To achieve this, we propose a CVAE-based model architecture. During training, the encoder processes both the input and response and leverages the recognition network to capture the potential variable *z*, which guides response generation and emotion recognition. To introduce perturbations, we incorporate a perturbation word selector to predict the type of each decoded word $y_{t}$ and determine whether to include a disturbance factor *r*. The global emotion label constraint, which utilizes an emotion classifier to identify the emotion of the hidden state $s_{t}$ during decoding, determines the value of the disturbance factor *r*. This ensures the generated response’s emotional consistency with the real response. To better learn the characteristics of the real response, we utilize KL divergence to close the gap between the prior network and the recognition network.

This paper’s contributions can be summarized as follows:

- A selective disturbance module is proposed that uses a disturbance word selector to perturb a portion of the response words based on learned potential variables, thereby improving response diversity.
- We introduce a global emotion label constraint to control the impact of perturbations during decoding, ensuring that the model improves response diversity while maintaining emotional expression.
- Our model’s ability to generate more diverse emotional responses compared to the baseline is demonstrated through extensive experiments on two standard datasets.

## 2. Related Works

### 2.1. Emotional Response Generation

In recent years, emotional dialogue generation methods have attracted significant interest. For instance, Zhou et al. proposed the emotional chat machine (ECM), which leverages external emotional vocabulary and internal emotional state memory to enable the system to generate responses of specific emotional categories [3]. Huang et al. utilized a special word that represents a specific emotion in a dictionary as an emotion marker at the encoder or decoder side. This pushes the decoder to generate responses with target emotions [13]. Song et al. proposed an emotion dialogue system (EmoDS) that leverages utterance-level classifiers and extra emotion vocabulary for generation [6]. Colombo et al. use a continuous representation of emotion to produce an emotional response in a controlled manner [14].

Using an emotion dictionary poses a challenge as the inclusion of fixed emotion words can result in a lack of consistency and diversity in the generated responses’ content. To address this issue, a CVAE-based emotion regularization method called Emo-CVAE has been developed to enhance the emotional expression of responses [15]. This approach greatly enhances the accuracy of predicting response emotions and also promotes diversity. However, Emo-CVAE only incorporates the emotion label as an additional input condition and does not explore the interplay between emotion and the content generated in responses.

Moreover, Rashkin et al. introduced the Empathetic Dialogues dataset, which was the first dialogue dataset focused on empathy [7]. It categorized dialogues into 32 emotional categories. In a similar vein, Lin et al. developed a specialized decoder that can generate responses tailored to the emotions expressed by the interlocutor [16]. Majumder et al. explored the concept of emotional imitation [17] and developed a generation model that relies on similar examples [18]. Subsequently, classical models emerged, such as the EmpTranfo model based on the GPT framework, which incorporates an empathy prediction task [19], and the CoMAE model, which employs a hierarchical approach to model empathy factors [20]. Nevertheless, these models have yet to achieve the ability to generate dialogue responses that authentically and accurately express emotions as humans do. In addition to empathic conversation generation, there are also studies from the perspective of emotion regulation that combine emotion and conversation intention to generate responses [21] together. The previous studies mentioned primarily emphasize enhancing the emotional representation of the model, but there is a lack of specific research on the diversity of the generated emotional responses.

### 2.2. Response Diversity

In Section 1, we mentioned that neural dialogue systems tend to produce high-frequency but boring responses. How to avoid this problem is a long-term problem in the research of response generation. Researchers have tried this in different ways. Some methods detail the training objectives of MLE loss [10,22,23]. Other methods directly design auxiliary loss terms to impose a certain penalty on the response [24,25]. In addition, alternatives to MLE are also tested constantly. Li et al. propose a diversity promotion goal based on maximum mutual information (MMI) [26]. On this basis, Zhang et al. propose to optimize with the objective of maximizing adversarial information [27]. Some researchers use constraints on target responses to enhance diversity [28,29]. An adaptive label smoothing method is proposed to adaptively estimate the distribution of targets in the processing of decode in different contexts [30]. Negative training strategies are also used to improve the diversity of responses [31]. Although these methods are effective to a certain extent, they also have some disadvantages. Take MMI as an example. Although it can lead to greater mutual information, the resulting response is likely to be the same in connotation as the input, and cannot bring more information. The possible reason is that the model can easily find a shortcut to achieve the maximum goal of mutual information by simply copying a portion of the markers in the last speech, rather than learning the conversational features.

Inspired by some related studies and combined with the idea of adversarial training [32,33], we apply certain disturbances to the process of response generation to make the model generate more diversified responses. It should be noted that dialogue models based on adversarial learning are difficult to train and may suffer from pattern breakdown, which is not conducive to a diversity of responses. Therefore, we choose to perturb the decoded word embedding rather than the decoded hidden state. At the same time, emotional labels of responses were used to constrain disturbances to ensure that increased diversity did not lead to decreased ability of emotional expression.

## 3. Approach

### 3.1. Formalized Definition

For a given input utterance $X=(x_{1},x_{2},\cdots ,x_{n})$, we aim to give the appropriate response $Y=(y_{1},y_{2},\cdots ,y_{m})$, and the response *Y* should have the appropriate emotion *e*, where *n* is the number of words in *X* and *m* is the length of the response, $e\in e_{1},\cdots ,e_{k}$, and *k* refers to the number of emotion categories. By connecting all the above inputs, we get the dialogue context c=[X;e]. Therefore, the target of response generation is

(1) $$P\left(Y,z|c\right)=P\left(z|c\right)\cdot P\left(Y|z,c\right),$$

where *z* is a latent variable for learning the characteristic distribution of the *Y*. P(z|c) means the sampling of *z* from the input, and P(Y|z,c) is the decoding process of generating the response according to the latent variables and context. It can be expressed as

(2) $$P\left(Y|z,c\right)=\prod_{t=1}^{m}P\left(y_{t}|y_{<t},z,c\right),$$

where $y_{t}$ is the decoding word at a current time step. $y_{<t}$ means the first t−1 words generated by the decoder.

### 3.2. Model Framework

**Figure 1:**
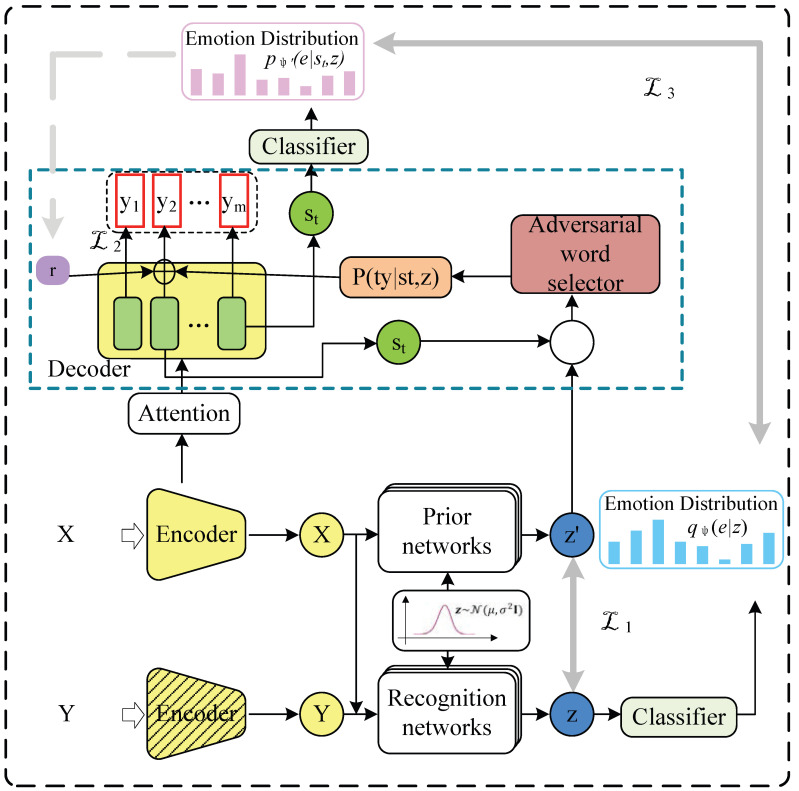
The total framework. During the training process, the input and real response are encoded respectively. The latent variable *z* is sampled by the recognition network, and a classifier is employed to identify the emotion category of *z*. In the decoding process, the perturbation word selector jointly predicts the type of the current generated word according to the hidden state $s_{t}$ and *z* and selectively applies the perturbation factor *r* to the decoding according to the type. At each time step of decoding, the currently generated response emotion is identified, and the disturbance factor *r* is dynamically constrained by bridging the gap between the emotion expressed in the generated response and the actual emotion. The section enclosed by the blue dotted line refers to the selective word disturbance module. The response encoder is only used for training purposes.

Our model overview is shown in Figure 1, which is built on the CVAE framework [34]. The encoder codes the input and response respectively and acts as the input of the identification network to obtain the latent variable *z*. The classifier performs emotion recognition for *z*. The perturbation word selector predicts the type of generated words $y_{t}$ according to z and decoder hidden state $s_{t}$ and controls the addition of perturbation. At the same time, emotion recognition is carried out on the decoder-generated response. By making the response’s emotion fit the real response’s emotion distribution respectively, the disturbance factor *r* is dynamically constrained, so that the generated response has similar emotion to the real response. The perturbation factor is applied to the word embedding of $y_{t-1}$ to influence the decoding, so as to achieve the goal of enhancing the diversity of response.

### 3.3. Basic Encoder-Decoder

Our model is implemented based on the Encoder-Decoder framework, and the basic Encoder-Decoder is introduced in this section. Here, $h_{t}$ is used to represent the current hidden state of the encoder and $s_{t}$ is used to represent the current hidden state of the decoder. The corresponding $h_{t-1}$ and $s_{t-1}$ represent the hidden state of the encoder and decoder at the previous time, respectively. The Encoder and Decoder can be specific structures such as RNN, LSTM, transformer, etc., so no specific network structure is used to refer to them.

For each word $x_{i}$ in input *X*, we first obtain its embedding representation $w(x_{i})$ and send it to the encoder. Then the hidden state $h_{t}$ is calculated by the current input $w(x_{i})$ and the previous hidden state $h_{t-1}$.

(3) $$h_{t}=Encoder\left(w\left(x_{t}\right),h_{t-1}\right).$$

To improve the performance of the decoder, dynamic attention is utilized to allow the decoder to focus on different content at different time steps prior to decoding

(4) $$a_{t}=\sum_{i=1}^{n}\left(\alpha_{i}^{t}h_{i}\right).$$

Here, $\alpha_{i}^{t}$ represents the weight between the decoder’s state $s_{t}$ and the encoder’s state $h_{i}$.

Based on these above, the decoder’s hidden state $s_{t}$ is updated by the previous hidden state $s_{t-1}$, dynamic attention $a_{t-1}$ and the previous generated word $y_{t-1}$. The softmax layer is hired to predict the current generated word $y_{t}$ by the decoder’s hidden state $s_{t}$

(5) $$s_{t}=Decoder\left(s_{t-1},a_{t-1},w\left(y_{t-1}\right)\right)$$

(6) $$y_{t}∼P\left(y_{t}|y_{<t},s_{t}\right)=softmax\left(Ws_{t}\right).$$

Here, $w(y_{t-1})$ represents the embedding of $y_{t-1}$.

### 3.4. Latent Variable Learning

Using the basic framework presented in Section 3.2 as a foundation, we incorporate two networks in CVAE, name recognition and prior, and conduct sampling of the input and response during both training and testing. The latent variable *z* contains rich features and plays a crucial role in the selection process of disturbance words and emotion classification of discourse.

We make the assumption that *z* follows a multivariate Gaussian distribution, with a diagonal covariance matrix. Specifically, in the process of training to identify the network response to real samples, we get a posterior probability distribution of $q_{\theta }\left(z|Y,c\right)∼N\left(\mu ,\sigma^{2}I\right)$. During the test process, the prior network $p_{\theta^{\prime }}\left(z|c\right)$ is used to extract the latent variable, which is involved in the response generation of the decoder. Obviously, the goal of the system is to make the generated response close to the real response. KL divergence is used to estimate the difference in probability distribution between the two. Minimizing the KL divergence between the prior network and the recognition network allows the former to better fit the latter. Therefore, we take the KL loss term as a part of the total system loss and write it as $L_{1}$. The parameterization of the above identification network and prior network can be achieved by MLP.

(7) $$\left[\mu ,\sigma^{2}\right]=MLP_{recog}\left(Y,c\right)$$

(8) $$\left[\mu^{\prime },\sigma^{\prime 2}\right]=MLP_{prior}\left(c\right)$$

(9) $$L_{1}=KL\left(q_{\theta }\left(z|Y,c\right)\|p_{\theta^{\prime }}\left(z|c\right)\right).$$

### 3.5. Adversarial Word Selector

Adding perturbations to models to enhance robustness has been practiced in some studies [32]. However, in the training of dialogue systems, the adversarial learning models are difficult to train and may have the problem of pattern collapse, so it is more inclined to generate boring responses. In order to address the issue of lack of diversity in the generated responses, we proposed a method to reduce the generation of high-frequency words in the model by introducing perturbations to influence the decoding process. The main aim of this method is to introduce disturbances to the generation of words during the decoding process, thus increasing the diversity of the generated responses. We introduce the emotional category of the response as a regulator. This means that the extent to which the disturbance affects the system’s output depends on the emotional category of the response. We use the emotional category as an auxiliary input to the decoder, which is concatenated with the decoder’s state and used to regulate the perturbation process. Specifically, we use a simple feedforward neural network to map the emotional category to a weight vector, which is then used to adjust the magnitude of the disturbance for each token in the response. This allows us to achieve a balance between diversity and emotional relevance in the generated responses. We use emotional labels to constrain the process, as described in Section 3.6.

It is important to note that not all generated words are suitable for perturbation. The research shows that topic headings play a very important role in dialogue interaction [35], and the random deviation of topics is not conducive to the continuity of dialogue. Therefore, our model needs to distinguish whether the current generated words are topic words or general words, to selectively perturb the decoding process. For the calculation of topic words, we refer to the PMI [36] method, that is, for any word $x_{i}$ in utterance *X* and $y_{j}$ in response *Y*, there is

(10) $$PMI\left(x_{i},y_{j}\right)=log\frac{p(x_{i},y_{j})}{p\left(x_{i}\right)p\left(y_{j}\right)}=log\frac{p(x_{i}|y_{j})}{p(x_{i})}.$$

PMI measures the co-occurrence of words in a corpus and can be used to identify words that frequently occur together. A higher PMI score indicates a stronger association between two words, which can be interpreted as them being more likely to be related to the main topic.

Further, we compute the PMI value between sequence $X=(x_{1},\cdots x_{n})$ and $y_{i}$. This means that each word in *X* is assessed for relevance to $y_{i}$, and a higher score can be interpreted that $y_{i}$ is more likely to be relevant to the topic.

(11) $$PMI\left(x_{1}\cdot \cdot \cdot x_{n},y_{j}\right)=log\frac{p(x_{1}\cdot \cdot \cdot x_{n}|y_{j})}{p(x_{1}\cdot \cdot \cdot x_{n})}$$

(12) $$\approx log\frac{\prod_{i=1}^{n}p\left(x_{i}|y_{j}\right)}{\prod_{i=1}^{n}p\left(x_{i}\right)}=\sum_{i=1}^{n}log\frac{p(x_{i}|y_{j})}{p(x_{i})}=\sum_{i=1}^{n}PMI\left(x_{i},y_{j}\right).$$

In the decoding progress, the adversarial word selector combines the current state $s_{t}$ and the hidden variable *z* to predict the category of the currently generated word. If it is a topic word, it will not be disturbed; otherwise, it will be disturbed.

(13) $$P\left(tp|s_{t},z\right)=softmax\left(W_{o}\cdot MLP_{adv}\left(s_{t},z\right)\right),$$

where $MLP_{adv}$ is the prediction network for the currently generated word class, $W_{o}$ is the corresponding weight matrix, and tp is the marker indicating whether the current word is the main topic, with values of 1 and 2.

### 3.6. Selective Adversarial Decoding

On the basis of the framework introduced in Section 3.2, we decode together with context, latent variables, and the prediction of adversarial word selector

(14) $$s_{t}=Decoder\left(s_{t-1},w\left(y_{t-1}\right),a_{t-1},c,z\right)$$

(15) $$\begin{array}{cc} P(y_{t}|y_{<t},c,z) & =P(y_{t}|y_{t-1},s_{t},c,z)\\ \; & =\sum_{i=1}^{2}P\left(tp=i|s_{t},z\right)P\left(y_{t}|y_{t-1},s_{t},c,z,tp=i\right), \end{array}$$

Here, tp=1,2 is the category of words, indicating whether the current generated word is a topic word, which is used to distinguish whether to add disturbance to the current word. The category is predicted by the perturbation word selector. When decoding $y_{t}$, we choose to add a disturbance to the embedding of $y_{t-1}$, rather than directly on the hidden state st, to ensure the independence of the disturbance effects when each response word is generated.

If tp=1, it means that the current generated word is a keyword, and its generation probability is

(16) $$P\left(y_{t}|y_{t-1},s_{t},c,z,tp=1\right)=softmax\left(W_{1}s_{t}\right).$$

Otherwise, it means that the current generated word is not a topic word, and a disturbance is added to $y_{t-1}$’s embedding.

(17) $$r=-\epsilon g/{∥g∥}_{2}$$

(18) $$g=\nabla_{x}logp\left(e|x;\hat{\theta }\right)$$

(19) $$w\left(y_{t-1}^{\prime }\right)=w\left(y_{t-1}\right)+r,$$

where *r* is the perturbation term added to the embedding of $y_{t-1}$, and *g* is the gradient of the emotional consistency loss (the loss is described in Section 3.7), in which *e* is the emotion category and $\hat{\theta }$ is the emotional classifier’s parameter set. The perturbation *r* uses $L_{2}$ normalization, which divides the value of each dimension of the gradient by its $L_{2}$-norm, in order to preserve the direction of the gradient. The prediction for the current word is represented by the following formula

(20) $$s_{t}=Decoder\left(s_{t-1},w{\left(y_{t-1}\right)}^{\prime },a_{t-1},c,z\right)$$

(21) $$P\left(y_{t}|y_{t-1},s_{t},c,z,tp=2\right)=softmax\left(W_{2}s_{t}\right).$$

Thus, the loss of the decoding process is

(22) $$\begin{array}{cc} L_{2} & =-E_{q_{\phi }\left(z|Y,c\right)}\left[ogP\left(Y|z,c\right)\right]\\ \; & =-E_{q_{\phi }\left(z|Y,c\right)}\left[\sum logP\left(y_{t}|y_{<t},z,c\right)\right]. \end{array}$$

### 3.7. Emotional Label Constraint

To prevent a significant deviation between the generated and actual response, it is crucial to control the amount of disturbance during the decoding process. To this end, we introduce a global emotional label constraint. In the training process, the recognition network obtains the hidden variable z, which is identified by the emotion classifier, so as to obtain the emotion distribution $q_{\psi }\left(e|z\right)$.

(23) $$q_{\psi }\left(e|z\right)=softmax\left(W_{E}\cdot MLP_{emo}\left(z\right)\right),$$

where $MLE_{emo}$ is an emotion classifier implemented by MLE, which identifies the emotion category of the real response according to the hidden variable *z*, and $W_{E}$ is the corresponding weight matrix.

When decoding, the current emotion type distribution $p_{\psi^{\prime }}\left(e|s_{t},z\right)$ is obtained by the emotion classifier under the influence of the hidden state st of the decoder and the hidden variable *z*. We expect the response generated by the perturbation decoding to be emotionally consistent with the real response. To limit the deviation between the generated response and the real response caused by excessive perturbation during the decoding process, we use a KL divergence to measure the distance between the two emotional distributions, and the perturbation signals *r* are constrained accordingly. The resulting loss of emotional restraint is

(24) $$L_{3}=\left(p_{\psi^{\prime }}\left(e|s_{t},z\right)\|q_{\psi }\left(e|z\right)\right)$$

### 3.8. Loss

The total loss of the model can be expressed as:

(25) $$L=\alpha L_{1}+L_{2}+\beta L_{3}$$

where $L_{2}$ is the reconstruction loss, $L_{1}$ is the KL divergence loss, and $L_{3}$ is the adversarial loss. α and β are hyperparameters that control the trade-off between the losses.

## 4. Experiments

### 4.1. Datasets

We use two datasets for our experiment, DailyDialog [37] and OpenSubtitles2018 [38]. **DailyDialog** contains ten topics and seven emotions, totaling 13,118 rounds of dialogues. The average conversation is 7.9 rounds and the utterances are 14.7 tokens on average. **OpenSubtitles2018** is a dialogue dataset from movie subtitles. The data set was filtered into conversations with sequences of 5–30 words long, each containing at least four utterances. The filtered data set contained 25,000 utterances.

Since the OpenSubtitles2018 dataset does not contain emotion labels, we need to train the dialogue emotion recognition model on other datasets to label the OpenSubtitles2018 dataset. IEMOCAP [39] and MELD [40] are two datasets commonly used in conversational emotion recognition tasks. Among them, IEMOCAP contains 151 dialogues with a total of 7433 utterances. Six types of emotions were labeled, among which non-neutral emotions accounted for 77%. MELD consists of 1433 dialogues and 13,708 utterances. The utterances in the dialogue were labeled with seven categories of emotions, of which 53% were non-neutral. It should be noted that IEMOCAP is played by professional actors, so emotions are expressed more clearly than in natural dialogue. The advantage of this dataset is its high quality and the limitation is its small data size. MELD, on the other hand, comes from the TV series Friends and several movies, and the dialogue is more natural. However, the dialogue in MELD involves too many plot backgrounds, so it is difficult to identify emotions.

In order to balance accuracy and generality, we trained several popular dialogue emotion classification models on two datasets, MELD and IEMOCAP. Since the sentiment categories of MELD and IEMOCAP are not exactly the same, we filtered the raw data to retain six sentiment categories shared by the two datasets. We selected the M2FNet [41] model that achieved the best performance on both datasets to label the OpenSubtitle2018 dataset. The relevant classification results are shown in Table 1. Note that M2FNet is a multi-modal dialogue emotion recognition model, but since OpenSubtitle2018 only contains text data, only text modal data are selected in our training and annotation.

### 4.2. Baselines

We select four related works as baseline models for comparison. They are CVAE [34], ECM [3], EMODS [6] and Emo-CVAE [15].

**CVAE** obtains a posteriori distribution of latent variable z in training based on seq2seq framework and uses prior distribution to fit a posteriori distribution in testing, so as to minimize reconstruction errors. **ECM** combines implicit internal emotional state changes with explicit external emotional vocabulary expressions to generate responses with specific emotions. **EmoDS** captures the emotional features of words and sentences to generate responses. **Emo-CVAE** introduces a conditional variational autoencoder model of emotion regularization, which is used to regularize the latent spaces of CVAE by adding an additional emotion recognizer.

In the interest of fairness, we implemented the basic modules of all the above models with GRUs (Bidirectional GRUs).

### 4.3. Settings

In the experiment, we used bidirectional GRUs to implement the encoder with a hidden size of 256, and 512 GRUs to implement the decoder. Pre-trained 300-dimensional word embedding [44] is employed for initialization. The dimension of latent variable *z* is set to 300. We used the ADAM optimizer [45]. The learning rate is 1 × 10${}^{-5}$, the batch size is set to 128, and the dropout rate is set to 0.2. A beam search with the size of 5 is used when decoding.

### 4.4. Automatic Evaluation

We use four automatic metrics, namely emotional accuracy (acc), dist-1, dist-2, and perplexity (ppl), for response evaluation in terms of emotional expression, diversity, and content. The accuracy of the emotion expressed by the generated response, or simply “Acc”, is used to evaluate the consistency of the emotion category between the generated response and the ground truth response. It measures the percentage of responses that are correctly classified into the corresponding emotion categories. **Dist-1/2** to evaluate the diversity, representing different single and double words in the generated responses. It is an evaluation at the n-gram level. **Ppl** stands for perplexity, which is commonly used to evaluate language models. It measures how well a model can predict a sequence of words in a given corpus. A lower perplexity score indicates that the model can predict the next word more accurately and with less uncertainty, which reflects a better-fitting ability of the model to natural language. In other words, the generated response content is relevant and syntactically correct.

Table 2 and Table 3 show the results of the automatic evaluation in DailyDialog and OpenSubtitles2018, respectively. ↑ represents that the larger the metric is better and ↓ means the opposite. The best results are shown in bold.

The results of the automatic evaluation showed that our model significantly improved the accuracy of emotional expression and the diversity of responses compared with the baseline method. In terms of confusion degree, ECM and EmoDS based on seq2seq generally performed better than other models based on CVAE. This makes sense because good diversity usually leads to increased confusion in the model. Compared with EmoCVAE, our model achieves better results in terms of emotional accuracy and response diversity and is almost the same in terms of perplexity degree.

**Figure 2:**
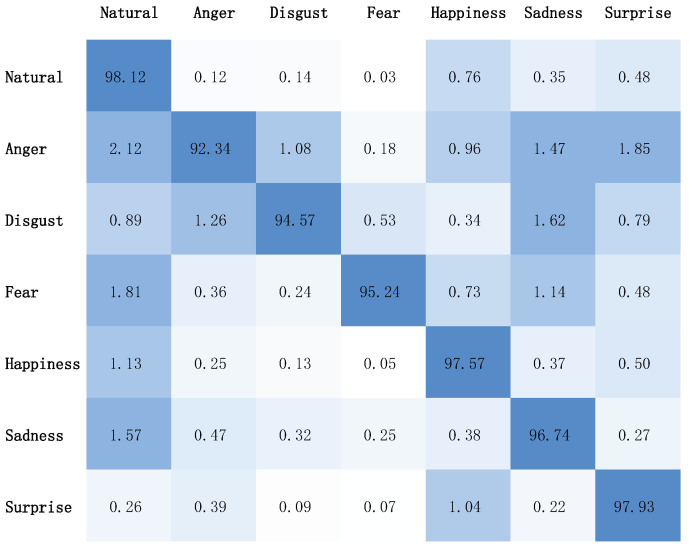
Confusion matrix of emotional classification on DailyDialog dataset.

In order to further study the performance of our model on emotional expression, the emotional accuracy of different categories in the DailyDialog dataset is given in Table 4. The corresponding confusion matrix of emotion classification is shown in Figure 2.

The results demonstrate that our model outperforms the baselines not only in terms of average performance but also in recognizing most emotion categories. This indicates that the global emotional label constraint proposed in our method has a positive effect on generating emotional responses. Notably, our model also achieves good performance for emotions that are difficult to identify by baseline models such as CVAE and ECM, such as anger and frustration. This can partially explain why our model is better at expressing emotions. Accurately identifying the emotion of the actual response is essential for accurately expressing the corresponding emotion.

As can be seen from Figure 2, our model tends to produce a relatively neutral response in more cases when the generated response emotion category is wrong (that is, inconsistent with the true response). In addition, our model is better at generating neutral, happy, and surprised responses than negative emotions such as anger and disgust. Our model is relatively less likely to generate responses where the emotion category is fear, possibly because it is the least represented in the original dataset.

### 4.5. Manual Evaluation

In addition to the above automatic evaluation, we also designed a manual evaluation to further verify the effectiveness of the proposed method. We used a pairwise comparison method to compare each of the four baselines.

The manual evaluation was conducted on the DailyDialog dataset, following the methodology of [15], which involves non-uniform random sampling to obtain samples based on the distribution of whether the emotion categories of responses generated by the baseline model are correct compared to ours. The evaluation uses the following notation: TT represents the responses with correct emotion categories generated by both our model and the baseline model, TF represents samples where the responses generated by our model have the correct emotional expression, but the baseline model does not, and FT and FF have similar meanings. The distribution of response samples is shown in Table 5.

For each case included in Table 5, 30 samples were randomly selected. We asked three evaluators to select responses that were better in terms of accuracy of emotional expression and variety of content. We allow ties to happen.

The results of the manual evaluation are shown in Table 6 and Table 7. By combining the results from the perspectives of sensibility and diversity, it can be seen that our model can generate more appropriate emotions under the three conditions of TT, TF and FF. Especially when our model correctly identifies the emotion of the response, the response produced by the decoder not only expresses the emotion better than the baseline models but also has certain advantages in terms of diversity.

It is also observed that when our model did not correctly identify emotion categories and when the baseline model correctly identified emotion in the case of FT, there was a significant decline in the dominance of the generated responses in terms of emotional expression. However, combined with the sample distribution in Table 5, the probability of this happening is very small, so it will not cancel out the advantages of our model in most cases.

### 4.6. Ablation Study

An ablation experiment was conducted on the DailyDialog dataset to verify the effectiveness of the proposed selective word perturbation module and global emotion control module. Two submodels were designed, one without selective adversarial (w/o SA), which does not add disturbance to the decoding process and only uses global emotion constraint to fit the emotion distribution of the generated and real responses. The other model adds fixed disturbances to non-subject words without using global emotion constraints and is denoted as w/o EC (without emotion constraint) only according to the perturbation word selector’s prediction. The DailyDialog dataset was used for the ablation study, and the experimental results were shown in Table 8.

The results of the ablation study demonstrate that when the model’s decoding process is not disturbed, its emotional expression ability remains largely unaffected. However, the diversity of the generated responses decreases significantly, and the performance is similar to that of the ECM baseline model. On the other hand, when the perturbation is not constrained, the diversity of the generated responses increases significantly, but the emotional expression ability and quality of the generated responses decrease significantly. This suggests that unconstrained perturbation is insufficient for generating high-quality responses. The selective perturbation and global emotional constraint modules proposed by our model are validated through the ablation study, showing their effectiveness in improving the diversity and emotional expression of the generated responses.

## 5. Conclusions

In this study, we propose a selective perturbation of emotional response generation to generate content-rich responses with appropriate emotional categories. The model is based on CVAE, and perturbation training is used to improve the diversity of response. In order to ensure that the dialogue topic does not have a large shift in the perturbation, we use the selective jamming module to predict the type of the current generated word according to the state and potential variables of the current decoder, so as to selectively apply interference to its decoding process. The global emotion constraint module uses the emotion distribution difference between the real response and the currently generated response to constrain the decoding interference, so as to ensure that the generated response is emotionally appropriate. Through the synergistic effect of the above two modules, the method proposed in this paper has achieved good results in the aspects of emotional expression and response diversity. Experiments on two standard datasets validate that our model outperforms baselines in generating more diverse responses with accurate emotions.

One potential direction could be to explore how this method could be adapted for use with pre-trained models, such as GPT-3 or BERT. Additionally, further research could be conducted to research the availability of this approach on other language generation goals beyond emotional response generation, such as machine translation or text summarization.

## Figures and Tables

**Table 1:** The performance of the emotion classifiers.

Method	MELD		IEMOCAP	
	Acc	wF1	Acc	wF1
DialogRNN [42]	59.54	57.03	63.40	62.75
DialogGCN [43]	59.46	58.10	65.25	64.18
M2FNet [41]	67.24	66.23	66.29	66.17

**Table 2:** Results of automatic evaluation metrics on the DailyDialog dataset.

Method	Acc ↑	Dist-1 ↑	Dist-2 ↑	Ppl ↓
CVAE [34]	82.53	0.074	0.358	65.79
ECM [3]	93.27	0.017	0.073	**62.31**
EmoDS [6]	88.06	0.013	0.049	63.68
Emo-CVAE [15]	95.14	0.083	0.407	64.52
Ours	**97.33**	**0.091**	**0.486**	64.72

**Table 3:** Results of automatic evaluation metrics on the OpenSubtitles2018 dataset.

Method	Acc ↑	Dist-1 ↑	Dist-2 ↑	Ppl ↓
CVAE [34]	79.62	0.072	0.403	67.29
ECM [3]	88.51	0.018	0.090	64.41
EmoDS [6]	86.27	0.012	0.037	**64.34**
Emo-CVAE [15]	90.34	0.096	0.512	66.38
Ours	**92.89**	**0.104**	**0.656**	66.21

**Table 4:** Results of emotional accuracy in different categories on the DailyDialog dataset.

Method	Natural	Anger	Disgust	Fear	Happiness	Sadness	Surprise	Average
CVAE [34]	96.29	49.27	74.19	83.85	90.16	81.79	85.33	82.53
ECM [3]	97.13	77.50	83.22	93.92	**97.62**	94.12		93.27
EmoDS [6]	95.92	62.31	65.98	92.03	97.27	90.08	95.87	88.06
Emo-CVAE [15]	97.35	91.06	**95.21**	93.99	96.90	95.28	96.13	95.14
Ours	**98.12**	**92.34**	94.57	**95.24**	97.57	**96.74**	**97.93**	**97.33**

**Table 5:** The distribution of response samples generated by baselines. The numbers in the table are percentages.

Method	TT	TF	FT	FF
CVAE [34]	80.69	16.22	2.39	0.70
ECM [3]	93.15	4.37	1.93	0.55
EmoDS [6]	85.43	16.86	1.89	0.82
Emo-CVAE [15]	94.72	3.81	1.26	0.21

**Table 6:** The performance of manual evaluation in terms of emotional expression (p<0.05).

	Win	Lose	Tie
vs. CVAE [34]			
TT	31.2	11.7	57.1
TF	64.3	4.5	31.2
FT	13.7	42.6	43.7
FF	35.9	8.4	55.7
vs. ECM [3]			
TT	29.6	22.5	47.9
TF	79.4	2.7	17.9
FT	12.2	54.3	33.5
FF	31.3	14.5	54.2
vs. EmoDS [6]			
TT	33.8	19.6	46.6
TF	72.5	2.9	24.6
FT	9.4	48.7	41.9
FF	30.6	12.3	57.1
vs. EmoCVAE [15]			
TT	24.9	19.3	55.8
TF	58.2	6.1	35.7
FT	9.1	43.4	47.5
FF	29.4	17.3	53.3

**Table 7:** The performance of manual evaluation in terms of diversity (p<0.05).

	Win	Lose	Tie
vs. CVAE [34]			
TT	35.8	21.5	42.7
TF	51.0	22.8	27.2
FT	35.4	26.7	37.8
FF	37.2	16.9	45.9
vs. ECM [3]			
TT	52.3	17.6	35.1
TF	43.9	28.5	27.6
FT	33.2	41.5	25.3
FF	26.6	32.7	40.7
vs. EmoDS [6]			
TT	45.6	19.2	35.2
TF	61.3	11.5	27.2
FT	52.9	31.7	15.4
FF	31.7	40.5	27.8
vs. EmoCVAE [15]			
TT	39.8	25.4	34.8
TF	47.6	12.3	40.1
FT	49.5	25.3	25.2
FF	33.3	26.7	40.0

**Table 8:** Results of ablation studies on the DailyDialog dataset.

Method	Acc ↑	Dist-1 ↑	Dist-2 ↑	Ppl ↓
Ours	**97.33**	**0.091**	**0.486**	64.72
w/o SA	97.82	0.018	0.092	64.25
w/o EC	83.90	0.121	0.706	**60.43**

## Data Availability

The two open access datasets DailyDialog and OpenSubtitles used in this study are available at http://yanran.li/dailydialog (accessed on 12 March 2020) and https://www.opensubtitles.org/ (accessed on 3 April 2020), respectively.
